# Supplementary material for: Effects of order on memory of event times
Source: Sci Rep. 2021 Aug 31;11:17456. doi: 10.1038/s41598-021-97032-w (PMC8408261; doi:10.1038/s41598-021-97032-w)
Supplement: Supplementary file 1 — Supplementary Information. [file 41598_2021_97032_MOESM1_ESM.pdf]

Supplemental material

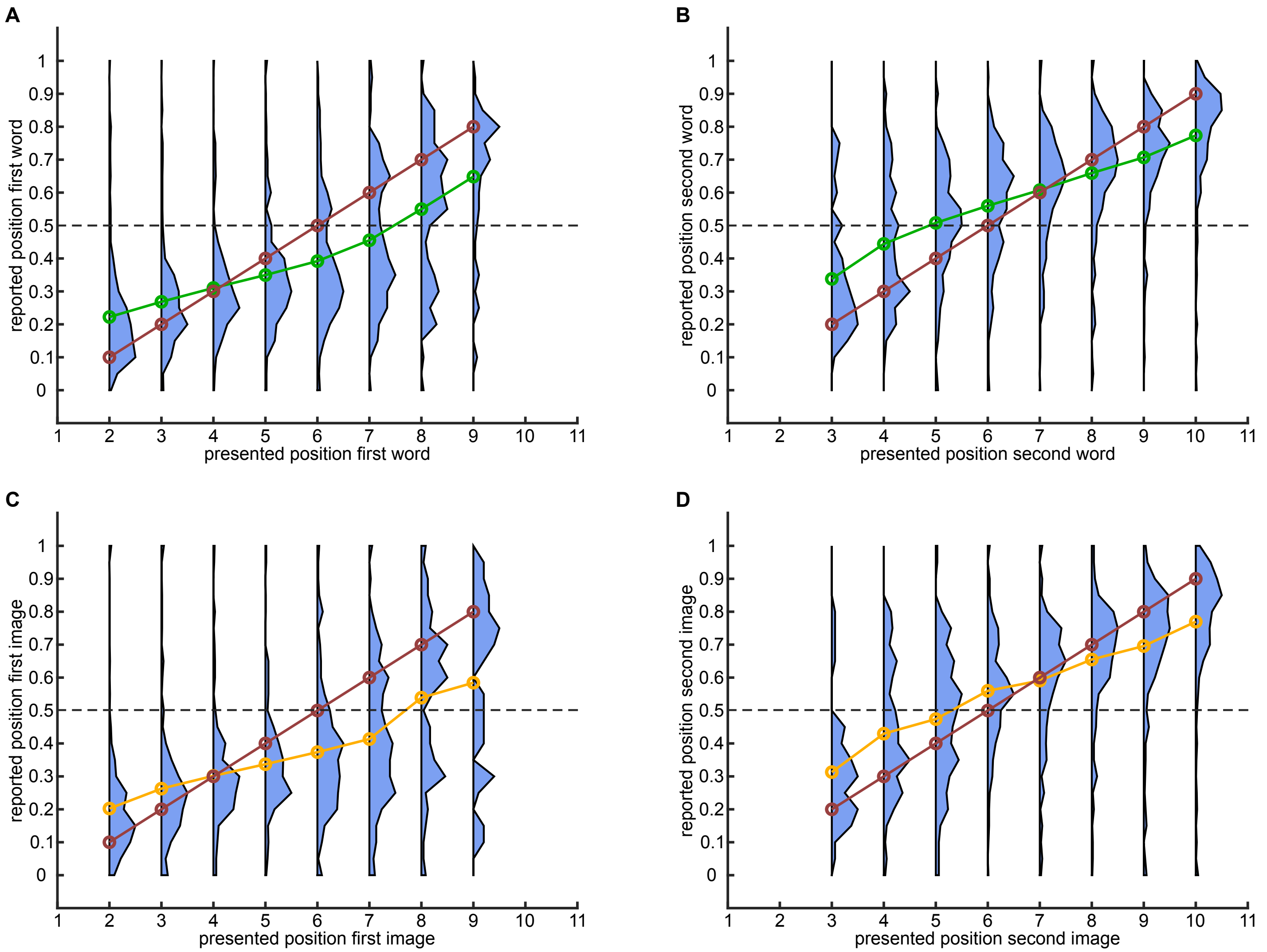

Figure S1: Experiment 2: distribution of reported times. **(A)**: For each presentation time of first intermediate word distribution of reported times. Green line corresponds to average of the distributions, red line corresponds to perfect report. **(B)**: Same for second intermediate word. **(C)**: Same for first intermediate image, where the orange line corresponds to average of the distributions. **(D)**: Same for second intermediate image.

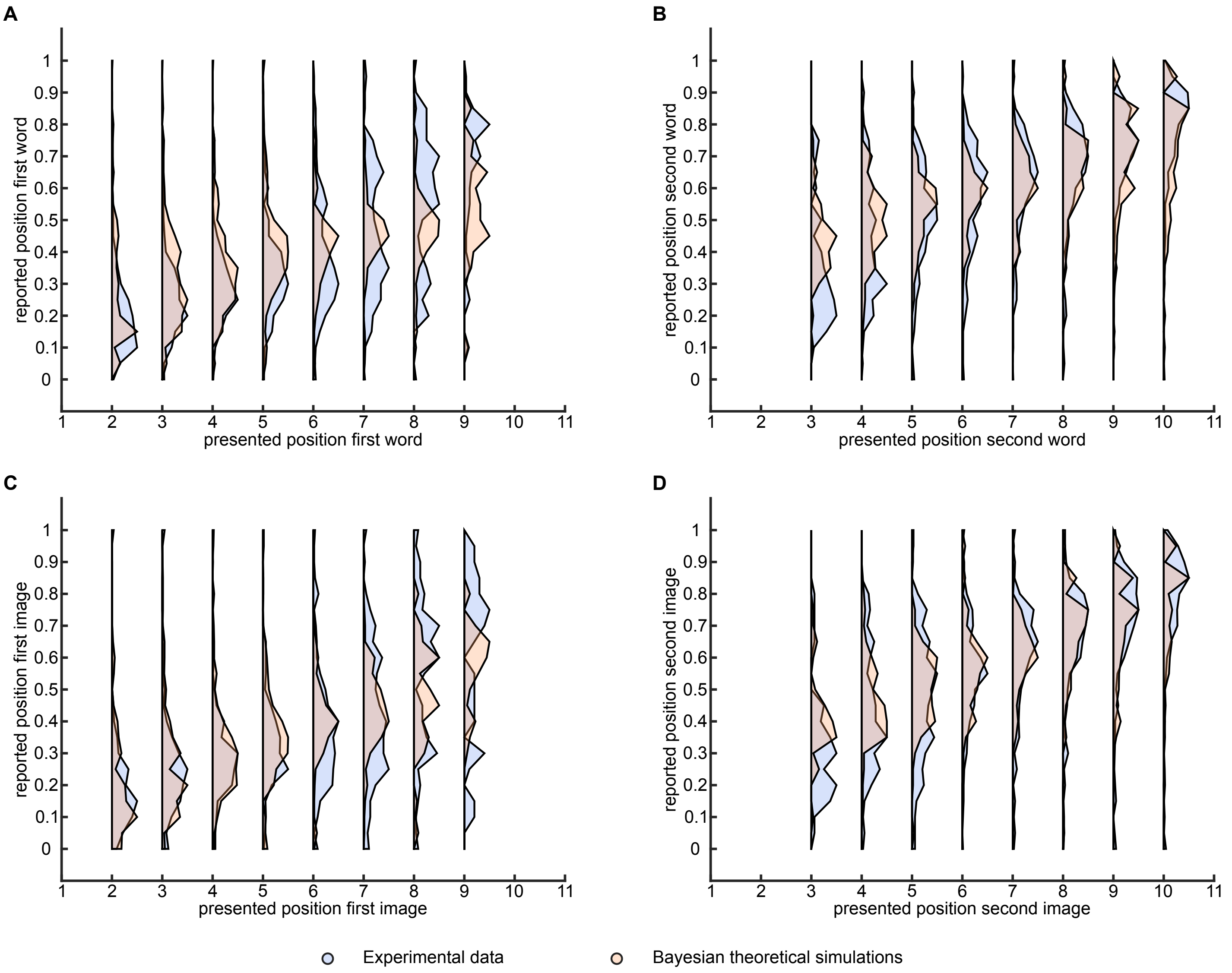

Figure S2: Comparison between Bayesian theory and 2nd experiment. **(A)**: For each presentation time of first intermediate word distribution of reported times. Blue corresponds to experimental data, while red to theoretical simulations. **(B)**: Same for second intermediate word. **(C)**: Same for first intermediate image. **(D)**: Same for second intermediate image.

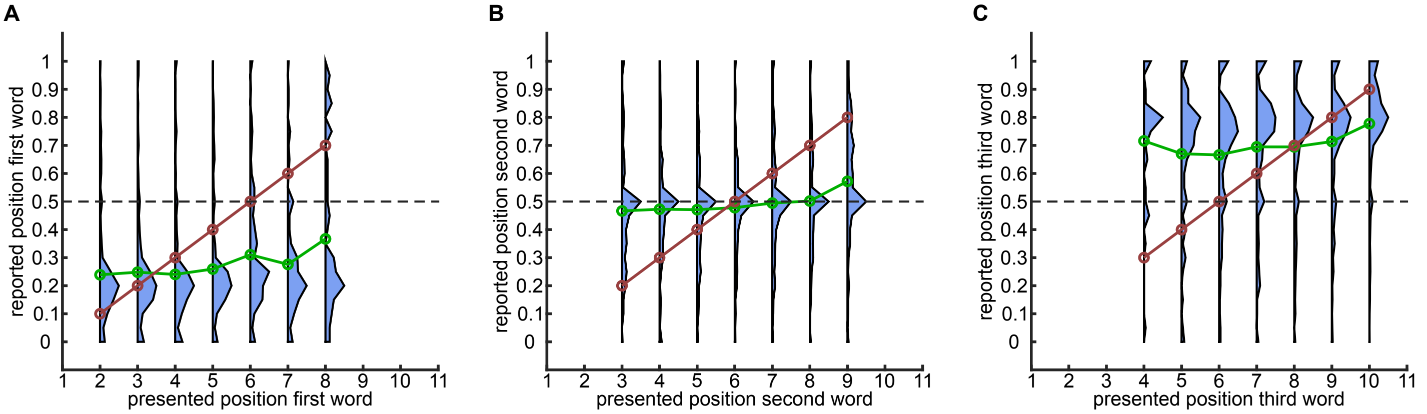

Figure S3: Experiment 3: distribution of reported times. **(A)**: For each presentation time of first intermediate word distribution of reported times. Green line corresponds to average of the distributions, red line corresponds to perfect report. **(B)**: Same for second intermediate word. **(C)**: Same for third intermediate word.

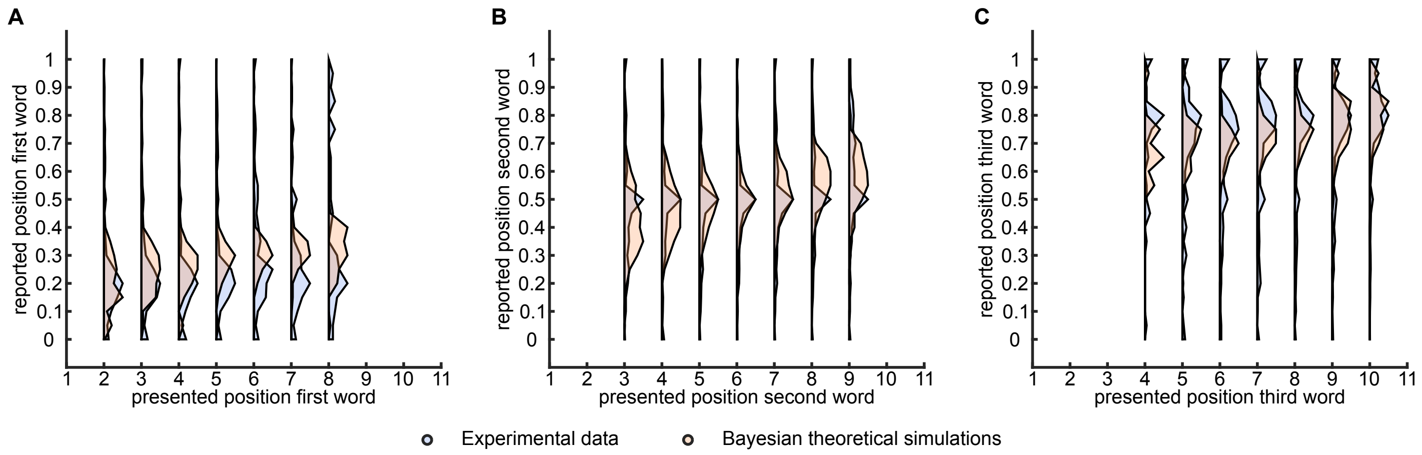

Figure S4: Comparison between Bayesian theory and 3rd experiment. **(A)**: For each presentation time of first intermediate word distribution of reported times. Blue corresponds to experimental data, while red to theoretical simulations. **(B)**: Same for second intermediate word. **(C)**: Same for third intermediate word.

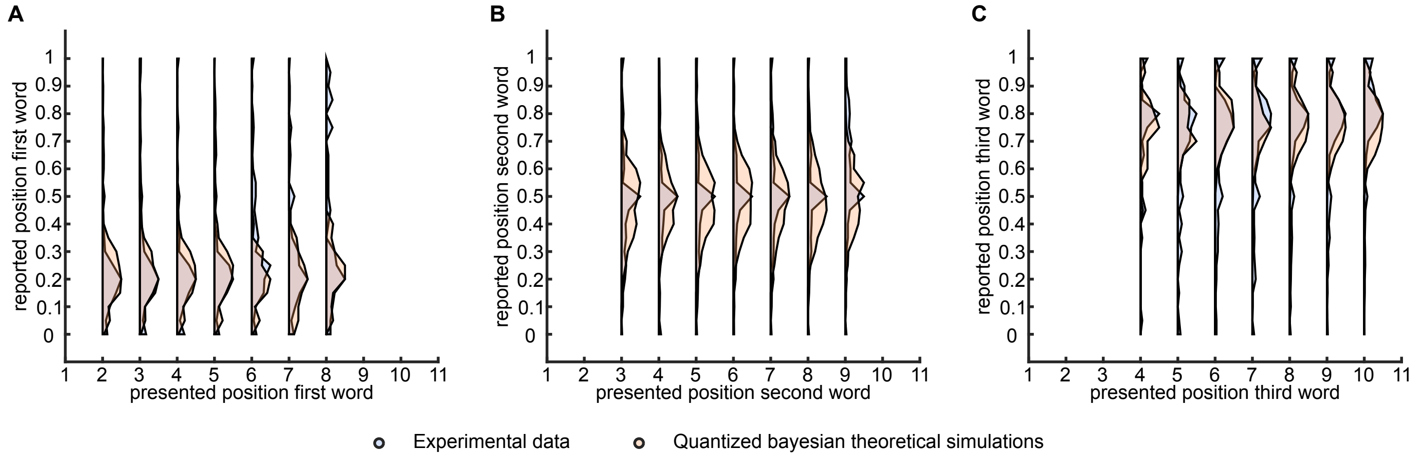

Figure S5: Comparison between Quantized Bayesian theory and 3rd experiment. **(A)**: For each presentation time of first intermediate word distribution of reported times. Blue corresponds to experimental data, while red to theoretical simulations. **(B)**: Same for second intermediate word. **(C)**: Same for third intermediate word.

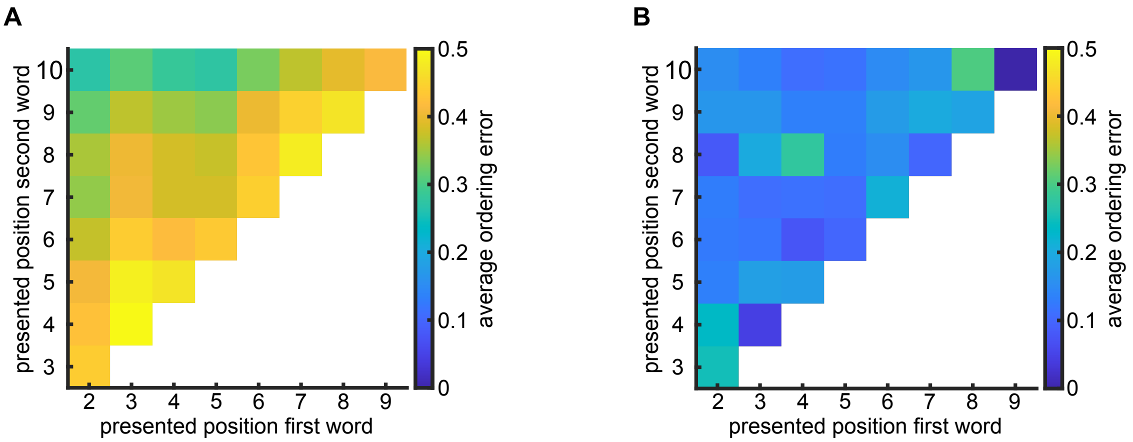

Figure S6: Experiment 4: Accuracy of relative time ordering with delayed reports filled with mathematical questions. **(A)**: Naïve prediction of average ordering error from independent distributions obtained with single intermediate word presented. Mean error is 40%. **(B)**: Experimental average ordering error with two presented words. Mean error is 15%.

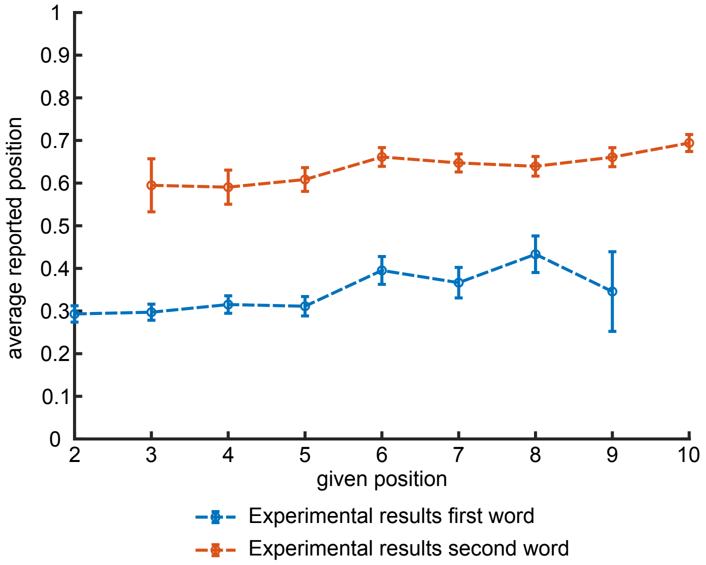

Figure S7: Experiment 4: average time reports. Average report times for first and second word, separately.
